# Influence of Different Amino Acids on the Aerosolization, Stability and Cytotoxicity of Spray-Dried Cannabidiol Dry Powder for Inhalation

**DOI:** 10.3390/pharmaceutics17091120

**Published:** 2025-08-27

**Authors:** Komal Komal, Lyall R. Hanton, Michelle Glass, Shyamal C. Das

**Affiliations:** 1 School of Pharmacy, University of Otago, Dunedin 9054, New Zealand; komko161@student.otago.ac.nz; 2 Department of Chemistry, University of Otago, Dunedin 9054, New Zealand; lhanton@chemistry.otago.ac.nz; 3 Department of Pharmacology and Toxicology, University of Otago, Dunedin 9054, New Zealand; michelle.glass@otago.ac.nz

**Keywords:** cannabidiol, lysine, arginine, cysteine, phenylalanine, dry powder

## Abstract

**Background:** Inhaled delivery of cannabidiol (CBD) through dry powder inhalers is a promising approach for achieving optimal drug concentrations in the lungs. Spray drying is a commonly employed technique to prepare inhalable powders with particle sizes ideally ranging from 1 to 5 µm, for deep lung deposition. However, formulating aerosolizable CBD dry powders remains challenging due to the thermolabile nature of CBD and the cohesive behaviour of micron-sized particles, which affects powder dispersibility, reduces de-agglomeration during inhalation, and causes inefficient lung deposition. These challenges can be overcome by the inclusion of excipients that can stabilize CBD during processing and enhance the dispersion and aerosolization of the powder. **Objectives and methods:** This study investigates the role of different amino acids (lysine, cysteine, arginine, and phenylalanine) in combination with inulin, a sugar-based excipient, on the in vitro aerosolization performance, stability, and cytotoxicity of inhalable CBD dry powders. **Results and conclusion:** The prepared CBD dry powders exhibited a size range of 1–5 µm. Amino-acid-free CBD powder showed an irregular and flaky morphology, while in association with amino acids, CBD dry powder showed spherical morphology with a dimpled surface. The ATR-FTIR spectra confirmed no interactions between CBD and amino acids in the dry powder formulations. CBD dry powder formulations containing amino acids demonstrated a better aerosolization profile compared to amino-acid-free CBD powder, with the lysine-containing formulation achieving the highest fine particle fraction (FPF) of 56.6%. Additionally, all the formulations were stable under low and high humidity (<15% RH and 53% RH) conditions for 28 days. Cytotoxicity studies on A549 alveolar basal epithelial cells showed that the amino acids were non-toxic, while the CBD formulations with/without amino acids showed comparable levels of cytotoxicity.

## 1. Introduction

Cannabidiol (CBD) is a non-psychoactive constituent of *Cannabis sativa*, garnering significant interest for its broad therapeutic properties. Preclinical and clinical studies have demonstrated therapeutic efficacy in alleviating symptoms associated with various conditions, including seizures, Parkinson’s disease, inflammation, cardiovascular diseases, rheumatoid arthritis, ulcerative colitis, chronic obstructive pulmonary disease, and asthma [1,2]. Currently, Epidiolex, an oral solution, is the only United States Food and Drug Administration (FDA) and European Medicines Agency (EMA) approved product available in the market [3,4,5]. It is indicated as an adjunctive therapy for seizures associated with Lennox–Gastaut syndrome (LGS) or Dravet syndrome (DS), in conjunction with clobazam, and tuberous sclerosis complex (TSC) for patients aged two years and older. However, orally administered CBD has shown low bioavailability and a variable pharmacokinetic profile due to its inherent characteristics, including low aqueous solubility, significant first-pass hepatic metabolism, and variable gastrointestinal absorption, resulting in suboptimal pharmacokinetics [6,7]. Given these limitations of oral CBD delivery, alternative administration routes have been explored to improve its systemic exposure and therapeutic efficacy. Inhaled drug delivery has garnered significant attention as a non-invasive route capable of achieving rapid systemic absorption and high bioavailability, thereby bypassing gastrointestinal degradation and hepatic metabolism. Compared to oral CBD solution, inhaled CBD dry powder had a 71-fold greater maximum plasma concentration and a 9.1-fold increased bioavailability in a Phase I clinical trial, highlighting the importance of inhaled drug delivery [8].

Among the different inhalation devices, dry powder inhalers (DPIs) are favoured over metered dose inhalers and nebulizers because of better product stability, easier administration, storage and transportation [9,10]. Effective lung deposition via DPIs requires particles having an aerodynamic diameter between 1 and 5 µm [11]. To achieve this, various formulation techniques have been utilized, such as spray-drying, ball-milling, spray-freeze drying, and thin-film freezing. Among these, spray-drying is the most extensively used technique for preparing inhalable dry powders, due to its capacity to prepare particles of the appropriate size and morphology and its scalability and reproducibility [12]. However, micron-sized spray-dried powders often exhibit interparticle cohesive forces, primarily driven by their high surface energy, morphology, and hydrophilic/hydrophobic properties [13,14]. These forces can negatively impact powder flowability, leading to inadequate flow from the capsule and de-agglomeration, thereby reducing aerosolization performance and lung deposition [15,16,17]. These challenges are especially pronounced in the case of lipophilic and thermolabile drugs such as CBD, requiring the incorporation of excipients to stabilize CBD and enhance powder dispersibility, stability, and aerosolization performance [18].

A range of excipients has been incorporated into CBD dry powder formulations to overcome these limitations, including sugars (mannitol, trehalose, lactose, and inulin (INU)), amino acid (leucine), phospholipids (dipalmitoyl phosphatidylcholine, and 1,2-distearoyl-sn-glycero-3-phosphocholine), polymers, cyclodextrins, magnesium stearate, and human serum albumin [8,19,20,21,22,23]. Among these excipients, INU, a naturally occurring oligosaccharide, was selected for this study due to its biocompatibility, non-toxicity, and multifunctional roles in stabilizing thermally unstable drug compounds [24,25,26,27,28,29,30]. Moreover, a recent study demonstrated that INU can stabilizes CBD during spray-drying [23].

Amino acids are generally used to enhance aerosolization and powder dispersibility due to their capacity to alter the physicochemical properties of dry powders [31]. Despite this, the use of amino acids as an aerosolization enhancer in inhalable CBD dry powder has only been minimally investigated [21,23]. To date, leucine is the only amino acid investigated for CBD dry powder formulations, where it has demonstrated promising results. For example, CBD dry powders prepared using thin-film freezing with 75% *w*/*w* leucine achieved a fine particle fraction (FPF) of approximately 84% whereas spray-dried CBD containing 20% *w*/*w* leucine exhibited an FPF of 62% [21,23]. These results indicate that the influence of amino acids on enhancing aerosolization depends on multiple factors, such as drug properties, the selection and concentration of amino acids. Various amino acids, including leucine [32,33,34,35,36], methionine [37], phenylalanine [13,32,37,38], asparagine [32], tryptophan [32,34,35,37,39], arginine [32,35,38,39], histidine [36], threonine [38], glycine [35,36], lysine [34], cysteine [40], and aspartic acid [35,38] have been investigated for their effects as aerosolization enhancers.

Moreover, the concentration of amino acids can substantially affect aerosolization performance [38,40]. However, the enhancement of aerosolization by amino acids is not universally observed across all drug formulations. For example, the incorporation of leucine into the spray-dried formulation of azithromycin did not significantly improve aerosolization compared to a formulation containing only azithromycin, with FPF of 62.5% and 62.9%, respectively [41]. Thus, screening a range of amino acids for their influence on aerosolization is a rational approach.

The functional properties of amino acids are formulation specific, so the present study evaluates the impact of four specific amino acids, lysine (LYS), cysteine (CYS), arginine (ARG), and phenylalanine (PHY), on aerosolization, stability and cytotoxicity of CBD dry powders containing INU as a primary excipient. The selected amino acids were chosen based on their physical characteristics, including hydrophobicity and surface activity [42,43,44,45].

## 2. Materials and Methods

### 2.1. Materials

Cannabidiol (CBD), (molecular weight 314.14 g/mol, and purity of 98.1%) was bought from Auxilio Pharmaceuticals, Auckland, New Zealand. Arginine (ARG), lysine (LYS), cysteine (CYS), phenylalanine (PHY), INU derived from chicory (CAT.I2255), and silicone oil were bought from Sigma Aldrich, St. Louis, MO, United States. High-performance liquid chromatography (HPLC)-grade solvents acetonitrile (ACN), pure ethanol (ETOH), and methanol (MeOH) were procured from Merck, Darmstadt, Germany. Milli-Q water was obtained from an in-house Millipore continuous water system (Millipore Corporation, Burlington, MA, USA).

### 2.2. Preparation of CBD Spray-Dried Powder Formulations

CBD dry powder formulations were prepared with a Buchi B-290 Mini Spray-Dryer (Buchi Labortechnik AG, Flawil, Switzerland). The spray-drying parameters were selected based on prior research [23]. Initially, CBD was spray-dried without excipients at a feed concentration of 0.2% *w*/*v*, which showed formulation challenges, resulting in low yield, significant adhesion to the drying chamber, and poor powder flowability. These findings emphasized the need for excipients to enhance processability and powder performance. The CBD concentration was fixed at 20% *w*/*w*, consistent with prior inhalation formulations that contain 20–25% CBD [8,20,21]. Similarly, amino acids were selected as 20% *w*/*w* corresponding to a 1:1 ratio with CBD, as previous studies demonstrated that this proportion enhances the powder flowability and aerosolization performance [37,38,46,47,48,49]. A total of 500 mL of feed solution was prepared at 0.2% *w*/*v* feed concentration based on the composition of Table 1. This lower feed concentration was selected because it results in a reduced geometric mean particle size, whereas higher feed concentrations are associated with the formation of larger particles due to increased droplet size and slower solvent evaporation during spray drying [50,51,52]. The preparation involved dissolving INU in hot water (60% *v*/*v*) (120 °C), cooling it to room temperature, and then adding amino acids. A separate CBD-ethanol (40% *v*/*v*) solution was prepared and mixed with the INU-amino acids solution. The final solution was spray-dried using fixed parameters from the previous study [23].

For each formulation, the CBD dry powder was collected, and the yield was calculated. The prepared CBD spray-dried powder was placed in a screw-top glass vial and kept in a desiccator at ambient temperature immediately after collection.

### 2.3. Drug Content and HPLC Method for Quantification of CBD

An HPLC method was used to analyze the CBD concentration in CBD spray-dried formulations. To prepare the solutions, 5–10 mg of CBD spray-dried powder was dissolved in 100 mL of a solution (60% ETOH and 40% water). The CBD concentration of each formulation was quantified using HPLC analysis, and all samples were analyzed in triplicate. An isocratic reverse-phase HPLC (Shimadzu, Kyoto, Japan) method was used to determine CBD concentrations based on a prior publication [53]. This analysis was performed on a C18 Synergi Fusion column (Phenomenex, Torrance, CA, USA), in which the mobile phase consisted of MeOH (85%) and water (15%). The oven temperature was set at 30 °C with a 1 mL/min flow rate. An injection volume of 20 µL was used for each sample, which was analyzed at a wavelength of 220 nm with a total analysis time of 12 min, with 6 min retention times. The calibration curves demonstrated high linearity (R^2^ > 0.999) across the 0.25–100 µg/mL concentration range.

### 2.4. Powder Physicochemical Characterizations

#### 2.4.1. Residual Solvent Content

Residual solvent content in the spray-dried powders was evaluated using a Q50 thermogravimetric analyzer (TGA) (TA Instruments, New Castle, DE, USA). A sample of 2–5 mg was placed on the TGA sample pan. The pan was then heated from ambient temperature to 120 °C at a rate of 10 °C per minute. The spray-dried powders’ weight loss was measured using TRIOS software (in-built software) to quantify the residual solvent content. Each formulation was tested in duplicate.

#### 2.4.2. Particle Size and Morphology Analysis

A scanning electron microscope (SEM) (Carl Zeiss Inc., Oberkochen, Germany) was utilized to evaluate the morphology of the supplied CBD, INU, LYS, CYS, ARG, PHY and prepared spray-dried powders. SEM can also measure the geometric particle size of inhaled powder [37,54]. Approximately 2 to 5 mg of raw material and prepared powder were distributed evenly on the surface of carbon adhesive tape and coated with gold/palladium alloy. SEM images of the prepared powders were acquired at an accelerating voltage of 5 kV. Particle size measurements were performed using ImageJ software (Version 1.53 k, National Institutes of Health, Bethesda, MD, USA) by analyzing around 300 particles [55,56].

#### 2.4.3. Drug-Excipient Interaction

The potential interaction between CBD and the excipients was examined using an Attenuated Total Reflectance–Fourier Transform Infrared (ATR-FTIR) instrument (Varian Inc., Palo Alto, California, CA, USA). Samples including CBD, INU, LYS, CYS, ARG, PHY and prepared spray-dried CBD dry powders were placed on the instrument’s crystal plate. Each spectrum was recorded using 64 scans at a fixed resolution of 4 cm^−1^ over a spectral range of 500–4000 cm^−1^. A Varian Resolutions programme (Varian, Palo Alto, California, CA, USA) was used to analyze the obtained spectra.

#### 2.4.4. Crystallinity of Powders

The crystallinity of supplied CBD, INU, LYS, CYS, ARG, PHY and prepared CBD spray-dried powder formulations was analyzed using powder X-ray diffraction (PXRD). T his analysis was performed using Cu Kα (λ = 1.54184 Å) radiation on an Agilent Technologies Supernova system. Finely ground powder samples were placed onto a nylon loop with a small amount of paratone-N oil. The data were collected at a 2θ range of 5° to 35° with a deviation of 0.010°. The data were analyzed using the CrysAlis Pro programme (version 1.171.35.15) [57].

### 2.5. In Vitro Aerosolization

The in vitro aerosolization performance of the prepared CBD spray-dried powder formulations was evaluated utilizing a Next Generation Impactor (NGI) (Copley Scientific Limited, Nottingham, UK). The prepared spray-dried powder was dispersed from a Foradil aerolizer (Novartis, UK) at a flow rate of 100 L/min, controlled by a Copley TPL 2000 critical flow controller and monitored using an electronic flow meter (Copley Scientific Ltd., UK). This flow rate created a pressure drop of approximately 4 kPa across the device, simulating a total inhaled volume of 4 L, representative of the typical inspiratory capacity of an average adult male [58]. To prevent the particles from bouncing, a few droplets of silicone oil (10^−5^ m^2^/s) were put on the NGI surface. An HPMC capsule (size 3, Capsugel, Tokyo, Japan) containing approximately 20 mg of powder was aerosolized for 2.4 s. The deposited powder was then dissolved in a solution of ETOH and water, and the solution was quantified using HPLC to measure the masses in the aerolizer, capsule, mouthpiece, induction port, NGI stages (S1–S7), and micro-orifice collector (MOC). The test was conducted in triplicate. Stages 1–7 of NGI have cut-off diameters of 6.12, 3.42, 2.18, 1.31, 0.72, 0.40, and 0.24 µm, respectively, at a flow rate of 100 L/min [48,59].

The in vitro aerosolization parameters include recovery dose (RD), emitted dose (ED), fine particle fraction (FPF), and fine particle dose (FPD). The RD indicates the total amount of drugs in the capsule, aerolizer, mouthpiece and induction port, and all stages of NGI, including MOC. The ED is the amount of drug emitted from the inhaler and deposited in the mouthpiece and induction port, NGI stages and MOC. The FPD is the amount of drug having an aerodynamic diameter of ≤5 µm after aerosolization. The FPD is determined by interpolating the graph of cumulative mass deposition at each NGI stage against the respective particle cut-off diameter [60,61]. The FPF is the ratio of FPD to ED.

### 2.6. Cellular Toxicity

The A549 human alveolar basal epithelial cell line (ATCC CCL-185^TM^, passages 94–99) was cultured in F-12K medium supplemented with 10% fetal bovine serum (FBS) and 1% concentration of penicillin solution (Gibco™ Antibiotic-Antimycotic (100×), Fisher Scientific, Hampton, NH, USA) a temperature of 37 °C and 5% carbon dioxide. Cell toxicity was evaluated using the Promega CellTiter 96^®^ Non-radioactive Cell Proliferation Assay (MTT, Promega Corporation, Madison, WI, USA). Cells were seeded into a 96-well tissue culture plate at a density of 50,000 cells per well. The prepared formulations were dissolved in dimethyl sulfoxide (DMSO). After a 24 h incubation period, the cells were subjected to treatment with vehicle control (medium + DMSO) or varying concentrations (1–60 µM) of raw materials, including CBD and spray-dried powder formulations, for a duration of 72 h [23]. Following the treatment period, 15 µL of MTT assay reagent was added to each well, followed by the incubation of the plates for 4 h at 37 °C with 5% carbon dioxide. After 4 h, stop solution was added, and absorbance was quantified at 570 nm utilizing a microplate reader (CLARIOstar Plus, BMG Labtech, Ortenberg, Germany). The log IC_50_ value was calculated by plotting cell viability against drug concentration using GraphPad Prism 10.4.2. The experiments were performed in three independent batches, each comprising three biological triplicates.

### 2.7. Stability Study

The effect of relative humidity (RH) on the prepared spray-dried CBD powders was evaluated by storing the powders in Petri dishes under two distinct conditions: low relative humidity (<15% RH) and high relative humidity (53% RH) at 25 ± 2 °C. The low and high humidity chambers were established using activated silica gel and a saturated magnesium nitrate solution [37,62]. After 28 days, the powders were collected and analyzed for in vitro aerosolization. Furthermore, their physicochemical properties were evaluated as described above.

### 2.8. Statistical Analysis

Statistical analysis was carried out using GraphPad Prism (version 10.4.2, USA). A one-way analysis of variance (ANOVA) was employed, followed by Tukey’s post hoc test. The significance level was set at *p* < 0.05. Results are expressed as mean ± standard deviation.

## 3. Results and Discussion

### 3.1. Yield and Physicochemical Characterizations of Spray-Dried CBD

The yield, particle size, residual solvent content, and drug content of the prepared CBD spray-dried powder formulations are presented in Table 2. The CBD spray-dried powder formulations with and without various amino acids were successfully prepared. The spray-drying parameters were selected based on the previous study [23]. The lower feed concentration was selected because it results in a reduced geometric mean particle size, whereas higher feed concentrations are associated with the formation of larger particles due to increased droplet size and slower solvent evaporation during spray drying [50,51,52]. The inlet temperature of 100 °C was selected after preliminary trials showed that temperatures below 90 °C did not produce dry powders but resulted in fused or sticky particles. At 100 °C, efficient solvent evaporation was achieved, and the resulting powders exhibited desirable morphology and dispersibility. This temperature was found to be optimal for the inulin–amino acid-based formulation system used in this study. The yield of the prepared dry powder formulation ranged between 5 and 55%, while CBD alone (C_SD_) had 5% yield due to CBD’s sticky and heat-sensitive characteristics, which pose challenges in preparing a dry powder. CBD’s known thermal instability is of particular concern during spray drying, as exposure to high inlet temperatures may lead to degradation. However, in this study, no signs of degradation were observed by HPLC or FTIR spectra (Section 3.3), indicating that the selected conditions were appropriate. The addition of INU and amino acids in the CBD formulation contributes to protecting CBD during the spray-drying process by creating a stabilizing matrix around CBD molecules, protecting them from thermal stress during atomization and drying. The addition of INU (CI_SD_) improved the yield to 29%, due to INU’s ability to stabilize the feed during atomization. The varying yields in different amino acid formulations can be linked to the physicochemical properties of amino acids. For example, formulations containing hydrophobic amino acids, such as PHY (CIP_SD_), achieved a yield of 55%. PHY has a relatively high hydropathy index, indicating low hydrophilicity, which may enhance its compatibility with the poorly water-soluble CBD in the spray-drying solvent system. The improved compatibility may facilitate more efficient solute-solvent interactions, leading to improved atomization and droplet drying, and consequently higher powder recovery. A similar finding has been reported in previous studies, where PHY contributed to a higher yield in dipyridamole formulations due to improved solubility characteristics [63]. However, it is important to note that a higher hydropathy index does not universally predict higher yield across all amino acids, as seen in the lower yield reported with other non-polar amino acids, such as CYS, in related studies. This suggests that factors beyond hydrophobicity, such as molecular structure and crystallization behaviour during drying, may also influence yield. The range of yield obtained in this (20 to 50%) is consistent with a prior study [64]. While amino acids generally enhance the powder yield, their impact depends upon multiple factors, including the type of drug, the concentration of excipients, and spray-drying parameters [36,38].

The residual solvent content in the prepared CBD dry powders remained below 5% *w*/*w* for dry powders, except for CI_SD_, which showed a relatively high value of 5.9% *w*/*w* [64]. This high residual solvent content can be due to the hygroscopicity of INU. Interestingly, incorporating amino acids into the formulations led to a decrease in residual solvent content, suggesting a moisture-modulating effect of amino acids. However, maintaining low residual solvent content is important since excessive residual solvent content can result in particle agglomeration, reduced flow, and affected aerosolization [65]. The lower residual solvent content indicates that the spray-drying process effectively removed excess solvent while maintaining the powders’ physical properties [66].

The concentration of CBD in the prepared spray-dried powder formulations was above 90%, aligning with the acceptable range recommended by the British Pharmacopeia [67].

The average geometric particle size of the prepared CBD spray-dried powder formulations ranged between 1.6 and 2.9 µm, with variations in particle size observed among different amino acid formulations (Table 2). The CIL_SD_ formulation showed the smallest particle size at 1.7 µm, while CIC_SD_, CIA_SD_, and CIP_SD_ had average geometric particle sizes of 2.6, 2.9, and 2.2 µm, respectively, with median diameter (D_50_) ranging from 1.5 to 2.7 µm. These results are consistent with our previous findings, where CBD-inulin-leucine formulations exhibited D50 values ranging from 1.3 to 1.8 µm for feed concentrations of 0.2% *w*/*v* [23]. These differences suggest that the type of amino acid added into the formulation influences the particle size due to their impact on the surface, droplet formation during spray-drying and particle aggregation behaviour [32,45]. The deposition of inhaled particles within the respiratory tract is predominantly influenced by their aerodynamic diameter. Particles with the aerodynamic diameter of 1–5 µm are ideal for deep lung deposition [68]. The aerodynamic diameter is related to the geometric particle size; generally, the smaller the geometric particle size, the smaller the aerodynamic diameter [68,69]. While care was taken to incorporate particles of all sizes, particle size determination from scanning electron micrographs is difficult for heterogeneous samples. For the prediction of particle behaviour during inhalation, particularly regarding deposition in the lower respiratory tract, particle size determination by laser diffraction, especially in the gas phase, would be advantageous.

### 3.2. Powder Morphology of Spray-Dried CBD

**Figure 1:**
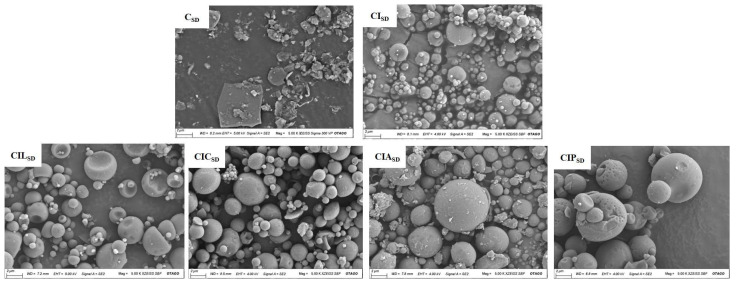
Representative SEM images of the prepared CBD dry powder formulations for CBD alone (C_SD_), CBD with INU (CI_SD_), CBD with INU and LYS (CIL_SD_), CBD with INU and CYS (CIC_SD_), CBD with INU and ARG (CIA_SD_), and CBD with INU and PHY (CIP_SD_).

The SEM images (Figure 1 and Figure S1) demonstrate the morphological characteristics of the prepared CBD spray-dried powder formulations and raw materials. Raw CBD exhibited an elongated, needle-like morphology, while the spray-dried CBD formulation (C_SD_) exhibited an irregular, flaky morphology. The inclusion of INU (CI_SD_) resulted in mostly spherical particles. However, noticeable aggregation was observed, which may be attributed to droplet adhesion to the dryer walls during the spray-drying process [70]. The addition of amino acids to the INU-CBD formulation maintained the overall spherical shape but introduced distinct differences in surface morphology. Specifically, the formulations containing LYS (CIL_SD_) showed a dimpled spherical morphology, while those with ARG (CIA_SD_) and CYS (CIC_SD_), exhibited a solid spherical shape. PHY (CIP_SD_) displayed spherical particles with a heterogeneous surface texture and occasional collapsed structures, possibly resulting from rapid moisture loss during drying and partial wall collapse.

Additionally, fine surface adhering particles were evident in CIL_SD_. These fine particles may arise from incomplete formation of smaller droplets or secondary nucleation during spray-drying [45,71]. To better understand the distribution and surface enrichment of amino acids on particle surfaces, analytical techniques such as X-ray photoelectron spectroscopy (XPS) or time-of-flight secondary ion mass spectrometry (ToF-SIMS) should be considered in future studies. Previous studies have reported variations in morphologies with different amino acids depending on the drug and processing conditions [38,40]. Dimpled spherical particles are advantageous for aerosolization, as they reduce particle aggregation and improve flowability and lung deposition [72,73]. Overall, particle morphology greatly influences aerodynamic behaviour by affecting drag forces and settling velocity, which in turn affect aerodynamic behaviour [74,75,76].

### 3.3. Drug Excipient Interaction of Spray-Dried CBD

**Figure 2:**
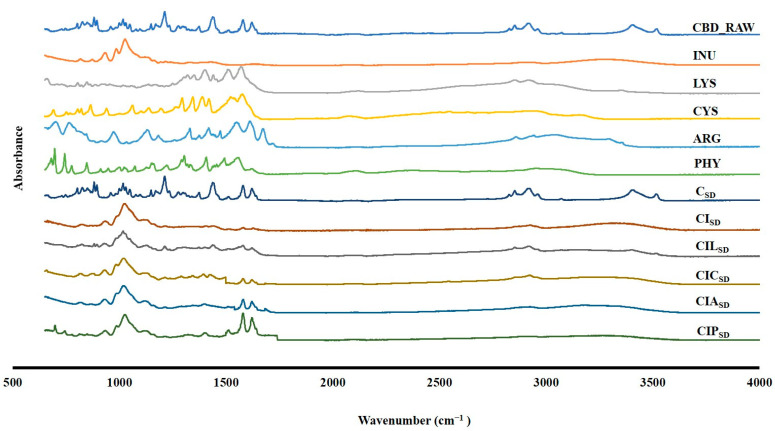
ATR-FTIR spectra of raw CBD (CBD_RAW), raw INU, LYS, CYS, ARG, PHY, and CBD dry powder formulations for CBD alone (C_SD_), CBD with INU (CI_SD_), CBD with INU and LYS (CIL_SD_), CBD with INU and CYS (CIC_SD_), CBD with INU and ARG (CIA_SD_), and CBD with INU and PHY (CIP_SD_).

The ATR-FTIR spectra of raw CBD, INU, CYS, ARG, PHY and prepared CBD spray-dried formulations are shown in Figure 2. The CBD spectra showed the principal peaks in the range of 1200 cm^−1^ to 1500 cm^−1^, indicating the presence of C-O bonds and the presence of alkene (C=C) bonds in the phenyl ring, respectively. Furthermore, hydroxyl stretching vibrations (O-H) were also present in the region of 3400–3500 cm^−1^, along with methine (C-H) groups in the phenyl structure [77]. INU and different amino acids had spectra similar to those published in the literature [78,79]. In the INU spectra, C-O-C-O-C stretching of the fructan ring and fructo-furanosyl group exhibited strong absorption in the range of 1000–1200 cm^−1^ [79]. However, the LYS spectra showed discrete peaks between 1400 and 1500 cm^−1^, indicative of CH_3_ and CH_2_ groups, whereas a broad absorption near 3000 cm^−1^ was associated with O-H and N-H stretching. Similarly, the CYS showed the presence of CH_2_ and CH_3_ groups from 1200 to 1400 cm^−1^. However, for ARG, the major absorption bands were present at 1633–1677 cm^−1^, indicating the presence of the CN_3_H_5_^+^ group. The band absorption for PHY was in the range of 1400–1660 cm^−1^, indicating the CC ring.

The spectra of prepared CBD spray-dried powder formulations showed no interaction between the drug and the excipients. However, INU peaks were more prominent in the CBD spray-dried powder formulations compared to those of CBD and different amino acids. The presence of CBD was confirmed by the detection of methyl and methylene groups at around 1500 cm^−1^. Variations in the peak intensities across different formulations may be attributed to factors such as uneven surface coverage, overlapping spectral contributions from excipients or potential physical interactions between excipients with CBD. Future studies, using techniques such as solid-state NMR or Raman spectroscopy, might provide further insights into these formulations, the structural integrity and molecular interaction.

### 3.4. Powder Crystallinity of Spray-Dried CBD

**Figure 3:**
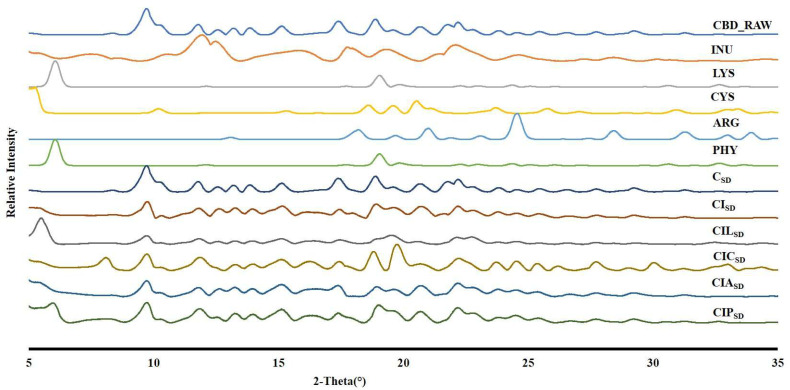
XRD spectra of raw CBD (CBD_RAW), raw INU, LYS, CYS, ARG, PHY, and CBD dry powder formulations for CBD alone (C_SD_), CBD with INU (CI_SD_), CBD with INU and LYS (CIL_SD_), CBD with INU and CYS (CIC_SD_), CBD with INU and ARG (CIA_SD_), and CBD with INU and PHY (CIP_SD_).

The PXRD spectra of the supplied CBD, INU, CYS, ARG, PHY, and prepared CBD spray-dried powder formulations are shown in Figure 3. The X-ray diffraction pattern of the CBD raw material showed sharp and distinct peaks, signifying its crystalline structure. Whereas the significant diffraction peaks were observed in the 2θ range of 5–30°, indicating the ordered molecular configuration of CBD [80]. However, the PXRD patterns of raw INU and amino acids showed sharp peaks, indicating their crystalline nature. The CBD spray-dried powder formulations exhibited a crystalline structure. Crystalline powders give better aerosolization and stability due to their lower surface energy and particle-particle interaction [81].

### 3.5. In Vitro Aerosolization of Spray-Dried CBD

**Figure 4:**
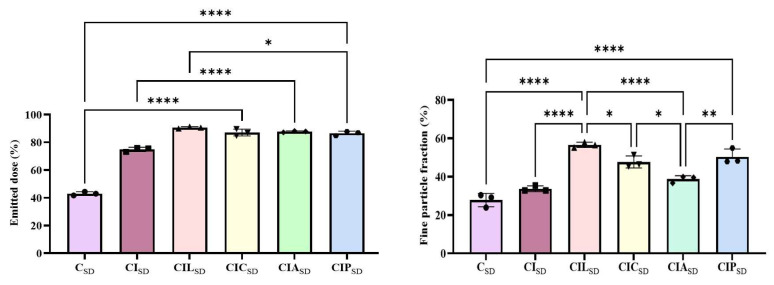
The in vitro aerosolization data of prepared CBD dry powder formulations for CBD alone (C_SD_), CBD with INU (CI_SD_), CBD with INU and LYS (CIL_SD_), CBD with INU and CYS (CIC_SD_), CBD with INU and ARG (CIA_SD_), and CBD with INU and PHY (CIP_SD_). Data represent the mean ± standard deviation for *n* = 3 (*, **, ****, statistically significant *p* < 0.05, < 0.002, <0.0001).

The in vitro aerosolization performance of CBD dry powder formulations with different amino acids is presented in Figure 4 and Table S2. Adding an amino acid resulted in enhanced aerosolization compared to the formulation of CBD alone or with INU (CBD-INU) (Figure S2 and Table S2). The recovery dose for all the formulations ranged between 92% and 97%. However, the ED for dry powder formulations was between 87% and 90% while for CBD alone it was 43%. Similarly, the FPF for the formulations varied between 38.7% and 56.6%, and it was 28% for CBD alone. Among these formulations, CBD dry powder with LYS (CIL_SD_), showed better FPF. The aerosolization of dry powder formulation is affected by several interrelated parameters, such as particle size, morphology, crystallinity of powder, surface energy, density, and hygroscopicity [37]. However, the exact mechanism of enhancing aerosolization by amino acids is not clear. It might be because amino acids reduce the surface energy, thereby decreasing interparticle cohesion and enhancing dispersibility, but this impact is significantly influenced by the drug-excipient interaction [37]. However, when we compared the aerosolization properties of the physical mixtures with spray-dried formulations, a significant difference was observed. Moreover, the FPF of the physical mixtures was approximately 20% (Table S2), showing low aerosolization compared to spray-dried formulations.

Comparable studies in the literature have reported wide variations in FPF values across different CBD dry powder formulations. For example, CBD with mannitol and DPPC with spray-freeze drying showed an FPF of 42%, compared to 33% with trehalose and DPPC [20]. CBD formulations with human serum albumin had an FPF of 30%, but 53%with methyl-β-cyclodextrin, despite utilizing the spray-freeze-drying method [82]. A spray-dried combination of 1,2-distearoyl-sn-glycero-3-phosphocholine (DSPC) and fumaryl diketopiperazine (FDKP) had an FPF of 30% [8]. Ball-milled formulations containing 2% and 5% magnesium stearate had FPFs of 27.1% and 30.6%, respectively [22]. These differences demonstrate the importance of excipient selection and particle engineering techniques.

### 3.6. Cellular Toxicity of Spray-Dried CBD

The cellular toxicity of raw CBD, INU, LYS, CYS, ARG, PHY, and prepared CBD spray-dried powder formulations was evaluated by measuring cell viability after exposure to varying concentrations of raw drug, excipients and prepared CBD spray-dried powders. The effects of CBD, INU, LYS, CYS, ARG, PHY, and prepared CBD spray-dried powder formulations on cell viability are shown in Table 3. Raw CBD exhibited cytotoxicity at high concentrations, while CBD spray-dried formulations had similar pIC_50_ values, suggesting that spray-drying and the addition of amino acids did not alter the cytotoxicity. None of the excipients (INU, LYS, CYS, ARG, and PHY) produced significant toxicity at concentrations up to 100 µM, indicating minimal cytotoxic effects. This confirms that the non-toxic excipients are suitable candidates for adding to inhalable formulations, as they do not contribute to additional cellular toxicity [83].

### 3.7. Stability Study of Spray-Dried CBD

A 28-day stability study was conducted at two different relative humidity (RH) conditions (25 °C, <15% RH (low humidity) and 25 °C, 53% RH (high humidity)). These conditions were selected to assess the effect of moisture on the physical stability of prepared spray-dried CBD dry powder formulations. Spray-dried formulations are susceptible to moisture, and exposure to humidity can induce physicochemical alterations that may affect their aerosolization performance. Therefore, evaluating the stability of these formulations under controlled RH conditions is important for predicting their long-term storage behaviour. These conditions do not fully align with International Council for Harmonisation of Technical Requirements for Pharmaceuticals for Human Use guidelines (ICH) guidelines and are considered preliminary screening parameters. Future studies will implement ICH-guidelines to confirm long-term stability. Four formulations, CIL_SD_, CIC_SD_, CIA_SD_, and CIP_SD,_ were selected for the stability study based on their superior aerosolization performance, as indicated by the highest FPF. Therefore, direct stability comparisons between amino acid-containing and amino-acid-free formulations were not conducted.

The prepared CBD spray-dried powder formulations exhibited no significant alterations in ED and FPF after 28 days, confirming that aerosolization performance was maintained (Figure S2). CBD showed better stability in powder form at ambient temperature. The morphological changes were observed in stored samples over time (Figure S3). Under low humidity conditions, all the formulations remain spherical. For CIL_SD,_ the appearance of more pronounced dimples on the surface during storage suggests partial recrystallization due to moisture uptake; in contrast, CIC_SD_ remains relatively spherical under both RH conditions. For CIA_SD_, increased surface irregularities and partial fragmentation were evident under higher humidity. CIP_SD_ showed no change in the morphology after 28 days in both conditions.

The ATR-FTIR spectra (Figure S4) confirmed the chemical stability of the prepared CBD spray-dried powder formulations. The spectra remained unchanged after storage, with no significant change in peak position, indicating that no chemical degradation occurred during storage. Similarly, with the XRD (Figure S5), patterns remained unchanged, showing the structural stability of formulations over time. Adding amino acids maintains CBD dry powders’ physicochemical stability under different storage conditions. The formulations’ drug and residual solvent content, as presented in Tables S2 and S3, showed no significant difference from day 0 to day 28 of the storage. However, a slight change was observed in the residual solvent content over this time, possibly due to moisture absorption under humid conditions.

## 4. Conclusion and Future Remarks

This study suggests that adding amino acids such as LYS, CYS, ARG, and PHY improves CBD dry powder formulations’ dispersibility and aerodynamic characteristics. LYS showed the highest FPF, attributed to its ability to modify particle morphology, resulting in improved aerosol performance.

Furthermore, all amino acids and INU were non-toxic to A549 alveolar epithelial cells, supporting their suitability as excipients for inhalable formulations. Adding amino acids to CBD dry powder formulations also reduced the cytotoxic effects of CBD, suggesting a potential protective role in cellular interactions. The prepared formulations showed stability under various humidity conditions, further confirming their suitability for long-term storage and clinical translation.

One limitation of the present study is the use of INU, which, although effective in enhancing powder yield and structural integrity, is not currently approved for inhalation. Its removal could increase the CBD loading capacity of the formulation. Future research should therefore investigate the feasibility of developing high-CBD-content powders using amino acids alone or in combination with excipients that are generally recognized as safe (GRAS) or approved for pulmonary administration, to improve clinical applicability.

Additionally, further research should focus on incorporating gas-phase aerosol characterization techniques to better assess the suitability of particles for deep lung deposition. Complementary studies should also include in vitro dissolution in simulated lung fluid, in vivo deposition, pharmacokinetic profiles, and therapeutic efficacy to further validate these formulations’ clinical applicability for treating pulmonary disease.

## Figures and Tables

**Table 1:** Compositions of the formulations.

Formulations	Concentration of CBD (% *w*/*w*)	Concentration of INU (% *w*/*w*)	Concentration of LYS (%*w*/*w*)	Concentration of CYS (%*w*/*w*)	Concentration of ARG (%*w*/*w*)	Concentration of PHY (%*w*/*w*)
C_SD_	100	-	-	-	-	-
CI_SD_	20	80	-	-	-	-
CIL_SD_	20	60	20	-	-	-
CIC_SD_	20	60	-	20	-	-
CIA_SD_	20	60	-	-	20	-
CIP_SD_	20	60	-	-	-	20

**Table 2:** Yield and physicochemical characterization of spray-dried CBD (mean ± standard deviation).

Formulations	Yield (%)	Particle Size (µm) *n*~300	D_50_ (µm)	Drug Content (%) *n* = 3	Residual Solvent Content (%) *n* = 2
C_SD_	5.4	1.7 ± 0.7	1.5	100.7 ± 1.7	0.12 ± 0.1
CI_SD_	29.7	1.6 ± 0.4	1.5	90.2 ± 1.1	5.9 ± 0.2
CIL_SD_	30.2	1.7 ± 0.7	1.5	94.5 ± 0.6	2.0 ± 0.0
CIC_SD_	26.6	2.6 ± 0.8	2.5	98.3 ± 0.3	1.6 ± 0.3
CIA_SD_	34.2	2.9 ± 1.1	2.7	98.7 ± 0.5	2.3 ± 0.0
CIP_SD_	55.6	2.2 ± 0.8	2.1	97.7 ± 0.8	1.8 ± 0.0

**Table 3:** In vitro cytotoxicity of prepared CBD spray-dried powder formulations.

Drug/Formulation/Raw Material	pIC_50_
Spray-dried CBD with lysine (CIL_SD_)	4.3 ± 0.5
Spray-dried CBD with cysteine (CIC_SD_)	4.3 ± 0.2
Spray-dried CBD with arginine (CIA_SD_)	4.3 ± 0.3
Spray-dried CBD with phenylalanine (CIP_SD_)	4.3 ± 0.5
Cannabidiol raw (CBD_raw)	4.4 ± 0.3
Inulin raw (INU)	>20
Lysine raw (LYS)	>20
Cysteine raw (CYS)	>20
Arginine raw (ARG)	>20
Phenylalanine raw (PHY)	>20

## Data Availability

Raw data can be requested by contacting the corresponding author.

## Supplementary Materials

The following supporting information can be downloaded at: https://www.mdpi.com/article/10.3390/pharmaceutics17091120/s1, Table S1. In vitro aerosolization data of CBD dry powder formulations and the physical mixture of the formulations. Figure S1. SEM images of raw CBD (CBD_RAW), raw lysine (LYS), raw cysteine (CYS), raw arginine (ARG), raw phenylalanine (PHY), and raw inulin (INU). Table S2. Drug content for 25 °C/<15%RH and 25 °C/53%RH for CBD dry powder with INU and LYS (CIL_SD_), CBD dry powder with INU and CYS (CIC_SD_), CBD dry powder with INU and ARG (CIA_SD_), and CBD dry powder with INU and PHY (CIP_SD_). Table S3. Residual solvent content for 25 °C/<15%RH and 25 °C/53%RH for CBD dry powder with INU and LYS (CIL_SD_), CBD dry powder with INU and CYS (CIC_SD_), CBD dry powder with INU and ARG (CIA_SD_), and CBD dry powder with INU and PHY (CIP_SD_). Figure S2. In vitro aerosolization of data of stability study; (A) emitted dose and fine particle fraction of day 0 and day 28 of 25 °C/<15%RH; and (B). 25 °C/<53% RH for CBD dry powder with INU and LYS (CIL_SD_), CBD dry powder with INU and CYS (CIC_SD_), CBD dry powder with INU and ARG (CIA_SD_), CBD dry powder with INU and PHY (CIP_SD_). Figure S3. SEM data for different relative humidity conditions, 25 °C/<15%RH and 25 °C/53%RH, for CBD dry powder with INU and LYS (CIL_SD_), CBD dry powder with INU and CYS (CIC_SD_), CBD dry powder with INU and ARG (CIA_SD_), and CBD dry powder with INU and PHY (CIP_SD_). Figure S4.ATR- FTIR for different relative humidity conditions, 25 °C/<15%RH and 25 °C/53%RH, for CBD dry powder with INU and LYS (CIL_SD_), CBD dry powder with INU and CYS (CIC_SD_), CBD dry powder with INU and ARG (CIA_SD_), CBD dry powder with INU and PHY (CIP_SD_). Figure S5. PXRD spectra for different relative humidity conditions, 25 °C/<15%RH and 25 °C/53%RH, for CBD dry powder with INU and LYS (CIL_SD_), CBD dry powder with INU and CYS (CIC_SD_), CBD dry powder with INU and ARG (CIA_SD_), and CBD dry powder with INU and PHY (CIP_SD_).
