# Supplementary material for: Influence of Different Amino Acids on the Aerosolization, Stability and Cytotoxicity of Spray-Dried Cannabidiol Dry Powder for Inhalation
Source: Pharmaceutics. 2025 Aug 27;17(9):1120. doi: 10.3390/pharmaceutics17091120 (PMC12473122; doi:10.3390/pharmaceutics17091120)
Supplement: Supplementary file 1 [file pharmaceutics-17-01120-s001.zip › pharmaceutics-3786470-supplementary.pdf]

pharmaceutics

MDPI

Supplementary Materials

Influence of Different Amino Acids on the Aerosolization, Stability and Cytotoxicity of Spray-Dried Cannabidiol Dry Powder for Inhalation

Komal Komal, Lyall R. Hanton, Michelle Glass and Shyamal C. Das

Table S1. *In vitro* aerosolization data of CBD dry powder formulations and the physical mixture of the formulations.

Formulations	Recovered dose (%)	Emitted dose (%)	Fine particle fraction (%)
C_{SD}	96.1 ± 1.4	43.0 ± 1.3	27.8 ± 3.4
CI_{SD}	94.2 ± 3.2	74.9 ± 1.5	33.7 ± 1.5
CIL_{SD}	97.4 ± 2.1	90.6 ± 0.7	56.6 ± 1.4
CIC_{SD}	95.9 ± 0.5	87.1 ± 2.4	47.7 ± 3.1
CIA_{SD}	91.2 ± 0.6	87.8 ± 0.5	38.7 ± 1.7
CIP_{SD}	92.0 ± 0.5	87.0 ± 1.5	50.7 ± 0.5
CIL_{PM}	94.3 ± 2.8	84.0 ± 2.0	20.5 ± 2.4
CIC_{PM}	94.6 ± 2.7	51.8 ± 3.0	6.7 ± 1.6
CIA_{PM}	90.0 ± 3.8	75.0 ± 0.7	23.4 ± 2.2
CIP_{PM}	98.2 ± 0.5	75.2 ± 1.9	20.1 ± 2.9

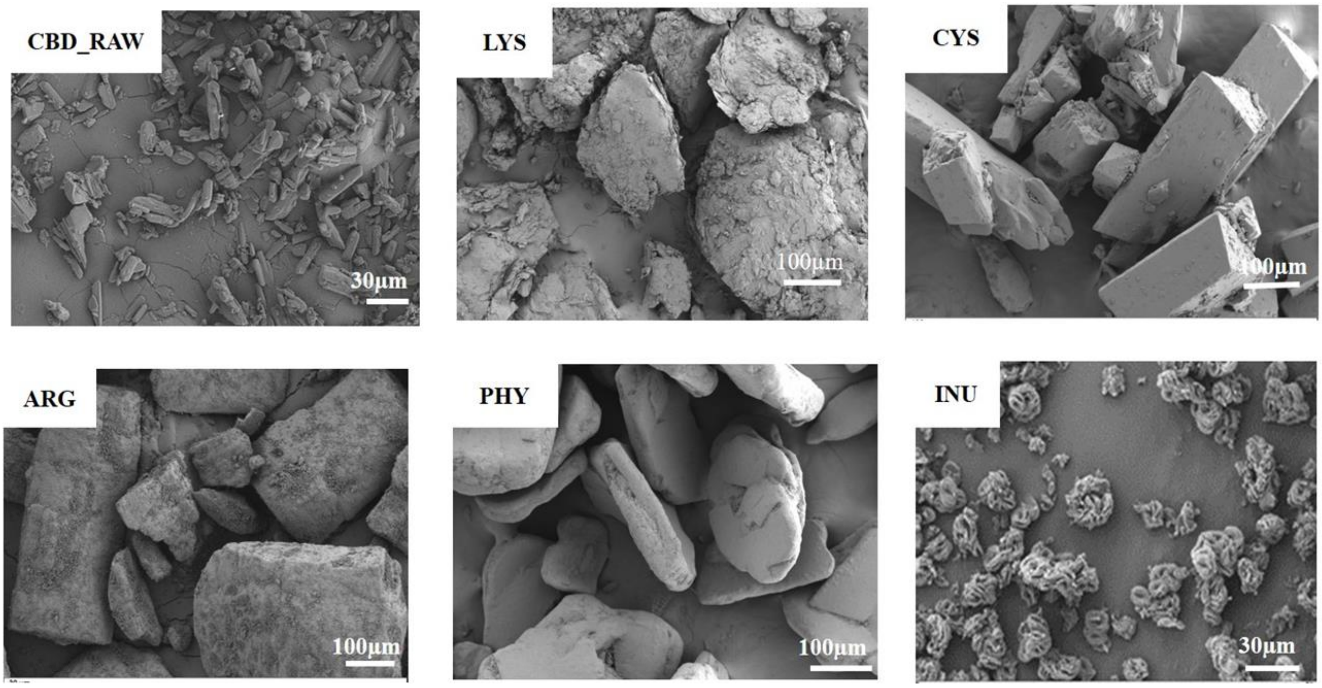

Figure S1. SEM images of raw CBD (CBD_RAW), raw lysine (LYS), raw cysteine (CYS), raw arginine (ARG), raw phenylalanine (PHY), raw inulin (INU).

Table S2. Drug content for 25°C/<15%RH and 25°C/53%RH for CBD dry powder with INU and LYS (CIL_{SD}), CBD dry powder with INU and CYS (CIC_{SD}), CBD dry powder with INU and ARG (CIA_{SD}), and CBD dry powder with INU and PHY (CIP_{SD}).

Formulations	Day 0	Day 28 (25°C/<15% RH)	Day28 (25°C/53% RH)
CIL_{SD}	94.5 ± 0.6	90.5 ± 1.4	90.7 ± 3.3
CIC_{SD}	95.9 ± 1.1	93.2 ± 1.2	90.2 ± 1.2
CIA_{SD}	98.7 ± 0.5	96.7 ± 0.6	94.7 ± 1.1
CIP_{SD}	97.7 ± 0.8	95.2 ± 0.2	93.2 ± 1.2

Table S3. Residual solvent content for 25°C/<15%RH and 25°C/ 53%RH for CBD dry powder with INU and LYS (CIL_{SD}), CBD dry powder with INU and CYS (CIC_{SD}), CBD dry powder with INU and ARG (CIA_{SD}), and CBD dry powder with INU and PHY (CIP_{SD}).

Formulations	Day 0	Day 28 (25°C/<15% RH)	Day 28 (25°C/53% RH)
CIL_{SD}	2.0 ± 0.0	3.4 ± 0.2	3.9 ± 0.2
CIC_{SD}	1.6 ± 0.1	3.2 ± 0.2	3.4 ± 0.3
CIA_{SD}	2.3 ± 0.0	2.7 ± 0.2	3.2 ± 0.3
CIP_{SD}	1.8 ± 0.1	2.6 ± 0.1	3.6 ± 0.2

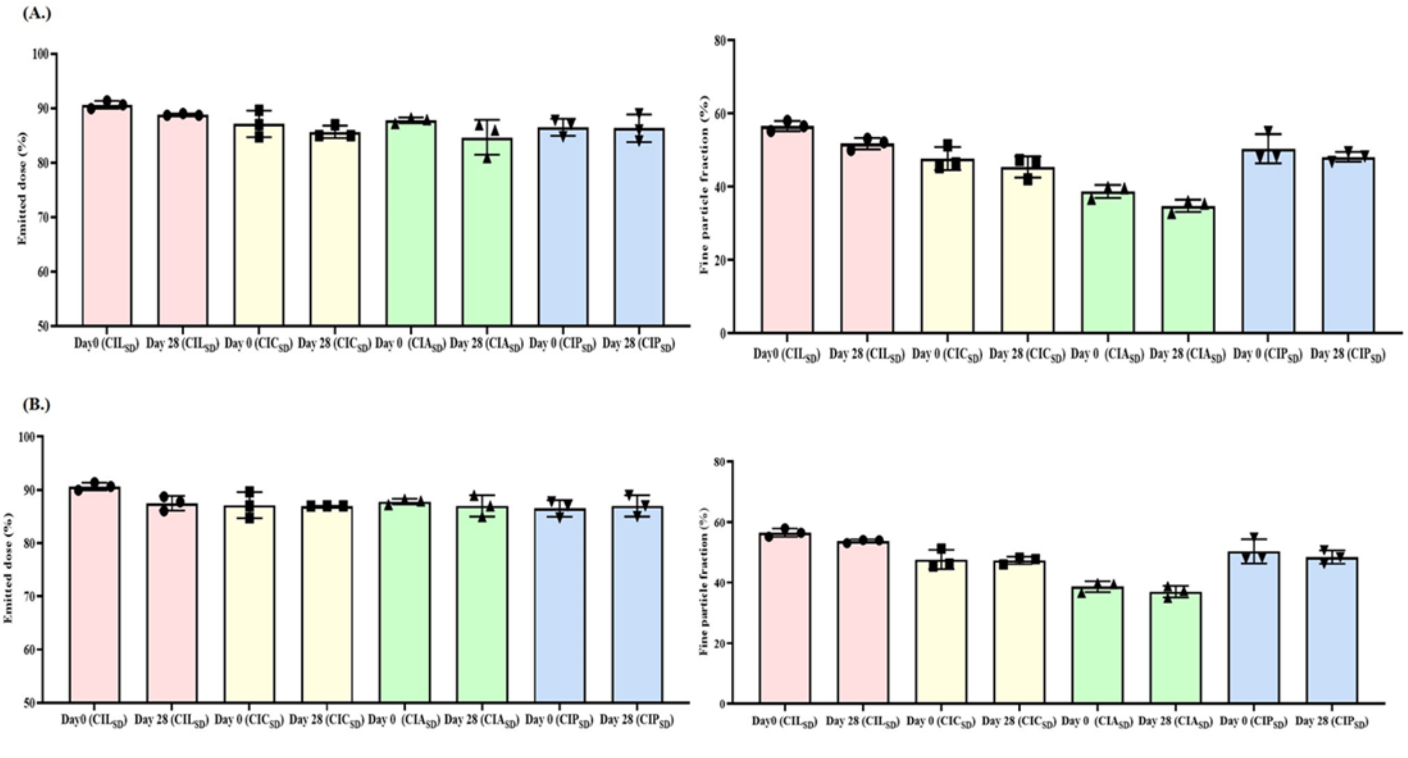

Figure S2. *In vitro* aerosolization of data of stability study**; (A.)** emitted dose and fine particle fraction of day 0 and day 28 of 25°C/<15%RH; and **(B).** 25 °C/ <53% RH for CBD dry powder with INU and LYS (CIL_{SD}), CBD dry powder with INU and CYS (CIC_{SD}), CBD dry powder with INU and ARG (CIA_{SD}), CBD dry powder with INU and PHY (CIP_{SD}). Data represent the mean ± standard deviation for n=3

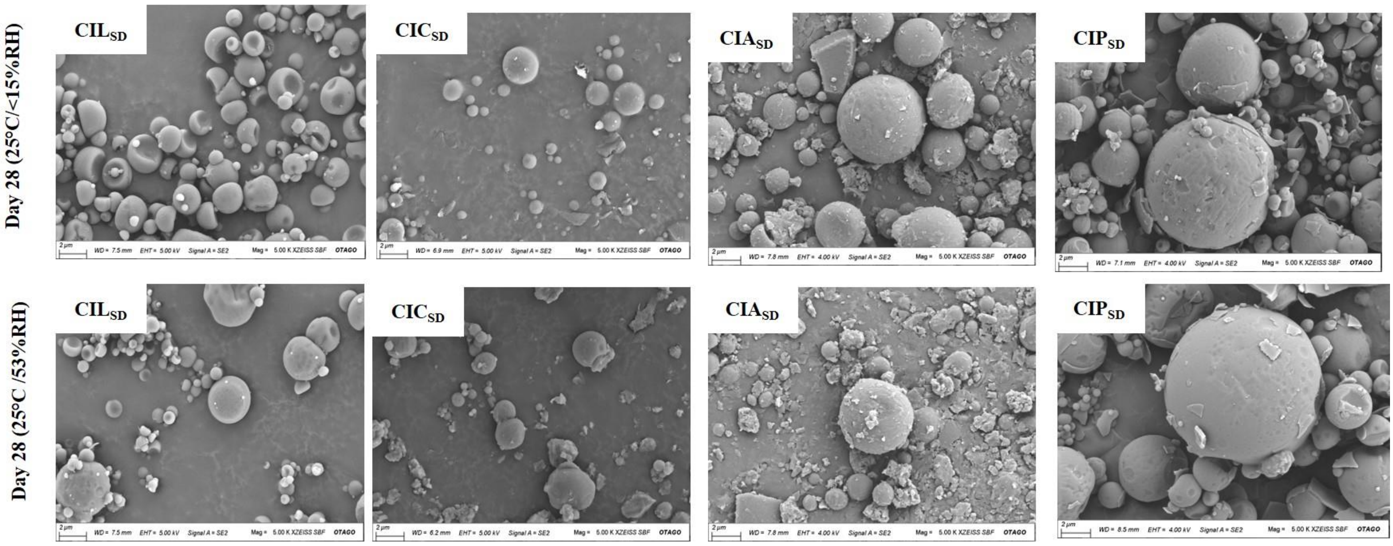

Figure S3. SEM data for different relative humidity conditions, 25°C/<15%RH and 25°C/ 53%RH, for CBD dry powder with INU and LYS (CIL_{SD}), CBD dry powder with INU and CYS (CIC_{SD}), CBD dry powder with INU and ARG (CIA_{SD}), and CBD dry powder with INU and PHY (CIP_{SD}).

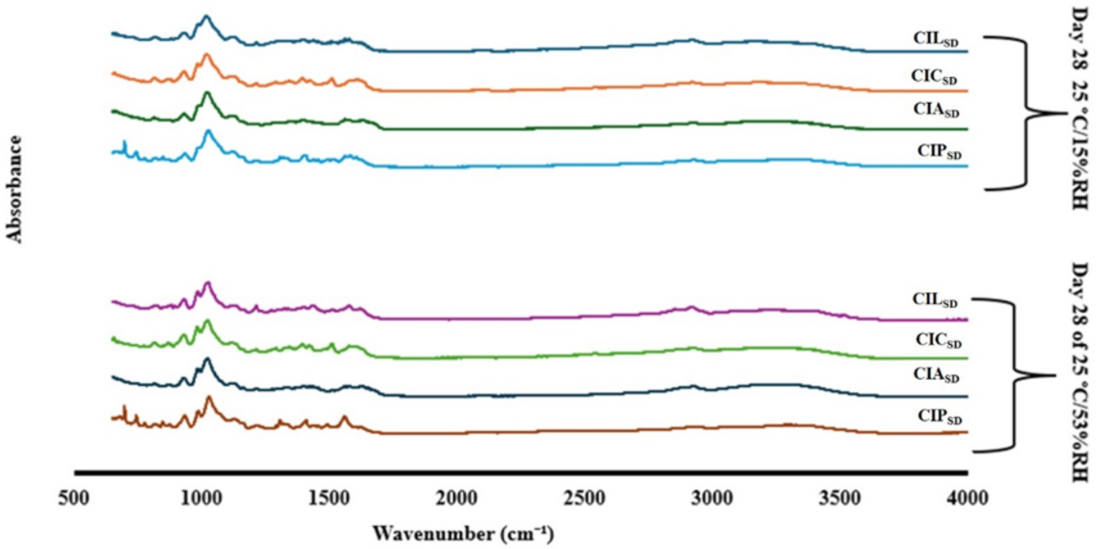

Figure S4. ATR- FTIR for different relative humidity conditions, 25°C/<15%RH and 25°C/ 53%RH, for CBD dry powder with INU and LYS (CIL_{SD}), CBD dry powder with INU and CYS (CIC_{SD}), CBD dry powder with INU and ARG (CIA_{SD}), CBD dry powder with INU and PHY (CIP_{SD})

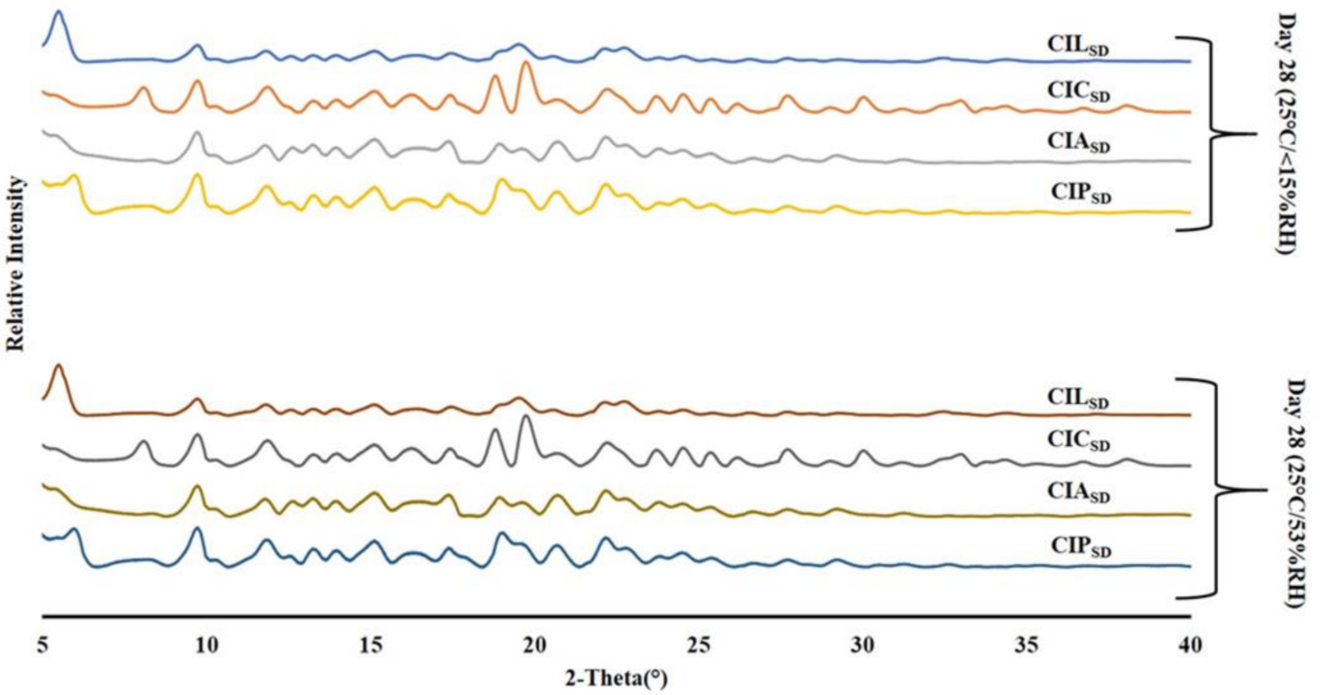

Figure S5. PXRD spectra for different relative humidity conditions, 25°C/<15%RH and 25°C/53%RH, for CBD dry powder with INU and LYS (CIL$_{SD}$), CBD dry powder with INU and CYS (CIC$_{SD}$), CBD dry powder with INU and ARG (CIA$_{SD}$), CBD dry powder with INU and PHY (CIP$_{SD}$).
